# Identification and characterization of flowering genes in kiwifruit: sequence conservation and role in kiwifruit flower development

**DOI:** 10.1186/1471-2229-11-72

**Published:** 2011-04-27

**Authors:** Erika Varkonyi-Gasic, Sarah M Moss, Charlotte Voogd, Rongmei Wu, Robyn H Lough, Yen-Yi Wang, Roger P Hellens

**Affiliations:** 1 The New Zealand Institute for Plant & Food Research Limited (Plant & Food Research) Mt Albert, Private Bag 92169, Auckland Mail Centre, Auckland 1142, New Zealand

## Abstract

**Background:**

Flower development in kiwifruit (*Actinidia *spp.) is initiated in the first growing season, when undifferentiated primordia are established in latent shoot buds. These primordia can differentiate into flowers in the second growing season, after the winter dormancy period and upon accumulation of adequate winter chilling. Kiwifruit is an important horticultural crop, yet little is known about the molecular regulation of flower development.

**Results:**

To study kiwifruit flower development, nine MADS-box genes were identified and functionally characterized. Protein sequence alignment, phenotypes obtained upon overexpression in *Arabidopsis *and expression patterns suggest that the identified genes are required for floral meristem and floral organ specification. Their role during budbreak and flower development was studied. A spontaneous kiwifruit mutant was utilized to correlate the extended expression domains of these flowering genes with abnormal floral development.

**Conclusions:**

This study provides a description of flower development in kiwifruit at the molecular level. It has identified markers for flower development, and candidates for manipulation of kiwifruit growth, phase change and time of flowering. The expression in normal and aberrant flowers provided a model for kiwifruit flower development.

## Background

Over the past decades, kiwifruit (*Actinidia *spp.) has developed into an important horticultural crop. The genus *Actinidia *belongs to the family *Actinidia*ceae within the Ericales order, contains 76 species originating mainly in China [1] and consists of perennial, climbing or straggling, deciduous plants. All members of *Actinidia *genus are functionally dioecious, with male and female flowers carried on different plants, typically at the basal end of the shoot [2]. Female flowers undergo androecial development but lack functional pollen and male flowers cease gynoecial development upon initiation of stigma. The reproductive cycles of kiwifruit commence after a juvenile period required for establishment of flowering competence. In mature kiwifruit plants, growth and flowering are spread over two growing seasons. During the first growing season, a number of phytomers and axillary meristems are initiated in latent shoot buds at the distal end of the shoot, which enter a dormant state and develop into inflorescence-bearing shoots early in the second growing season, at spring budbreak [3-7]. Kiwifruit inflorescences are compound dichasia, but lateral flowers in most female cultivars cease development soon after their initiation and only terminal flowers develop [8].

Conflicting reports are available on the timing of floral commitment, ranging from the spring of the first growing season [4,5,9] or late summer of the first growing season [10], to the spring of the second growing season, immediately before flower differentiation [11]. In addition, flower development during the second growing season depends on environmental conditions, most importantly winter chilling; insufficient chilling results in unsynchronized budbreak, low flower numbers and subsequent low fruit yields. *Actinidia *species differ significantly in their timing of budbreak, winter-chilling requirements, lengths and numbers of nodes per shoot, indicating a genetic control of shoot growth and flowering.

Current research on kiwifruit is mainly focused around consumer-driven traits such as fruit flavour and fragrance, appearance, healthful components and convenience [12], but the knowledge of the genetic regulation of growth and development, flowering and sex-determination is very scarce, yet essential to accelerate breeding and aid our understanding of flowering control in kiwifruit and woody perennial species in general.

Molecular and genetic regulation of flower development has been subject to detailed analysis in various plant species. Specification of floral organ identity in model plants *Arabidopsis *and *Antirrhinum *has been explained by the classical ABC model [13,14]. Identity of floral organs is determined by three classes of function, A, B and C, each consisting of one or more genes [15-27]. Further research resulted in the revised ABC(D)E model [28-34]. In addition, the A class gene *APETALA1 *(*AP1*), together with other members of the *AP1/FUL*-like gene family and *SEP *gene family, have a role in specification of floral meristem identity [35,36]; another A class gene *APETALA2 *(*AP2*) is also implicated in the control of floral transition [25], seed size [37] and maintenance of the stem cell niche in the shoot meristem [38]. With the exception of *AP2*, all the floral organ identity genes are members of the MADS-box family [reviewed in 39]. They all belong to the plant-specific MIKC type MADS-box genes [40], orthologs from different plant species generally belong to the same MADS-box gene subfamilies [41-50] and their function is well correlated with expression patterns [51].

In general, the ABC and ABCE models are widely applicable to non-model plants, with a few caveats. Whereas the B, C and E functions are regarded to be broadly conserved, the A function in specification of the perianth is not widely observed and questioned in *Antirrhinum *[52,53], as well as other plants [54]. In addition, this model fails to explain floral diversity seen within flowering plants, and additional models have been proposed [55-57]. Evolutionary developmental biology of MADS box genes in a range of angiosperms has been instrumental in the development and testing of these models [reviewed in 58] and further broad comparative studies, including normal and aberrant flowers in a range of species, will aid understanding of the mechanisms underlying the variation in angiosperm floral morphology.

The objective of this study was to functionally characterize genes required for development of kiwifruit flowers. Specifically, this study aimed to: (i) identify genes that specify floral meristem and floral organ fates in kiwifruit; (ii) identify if specific expression patterns may have led to the aberrant morphology of some kiwifruit flowers; and (iii) develop molecular markers to monitor kiwifruit floral development. Nine MADS-box genes highly similar to class A, B, C, and E function genes were identified and further characterized using cultivars of the closely related kiwifruit species, *A. chinensis *and *A. deliciosa *and an *A. deliciosa *spontaneous mutant 'Pukekohe dwarf' with an abnormal floral phenotype. We discuss kiwifruit flower development in the light of the existing flowering models.

## Results

### Identification of kiwifruit candidate genes

Nine non-redundant kiwifruit MADS-box genes were identified on the basis of similarity to *Arabidopsis *floral MADS-box genes, and named *Actinidia FUL-like*, *FUL*, *AP3-1*, *AP3-2*, *PI*, *AG*, *SEP1*, *SEP3 *and *SEP4 *(Table 1). For some genes, multiple near-identical sequences were recovered reflecting alleles, sequences from different genomes within polyploid genomes or orthologs from different kiwifruit species (Table 1).

**Table 1:** *Actinidia *flowering genes

**Gene name**	**Total ESTs**	**Kiwifruit species**	**Organ/tissue**	**GenBank accession number**
*FUL-like*	1	*A. chinensis*	young leaf	HQ113356

*FUL*	10	*A. chinensis A. arguta*	young fruit, ripe fruit	HQ113357

*AP3-1*	8	*A. chinensis, A. arguta*	ripe fruit, petal	HQ113358

*AP3-2*	4	*A. eriantha, A. deliciosa*	petal	HQ113359

*PI*	5	*A. polygama*, *A. arguta*	petal	HQ113360

*AG*	2	*A. arguta*	ripe fruit	HQ113361

*SEP1*	1	*A. chinensis*	young fruit	HQ113363

*SEP3*	4	*A. chinensis*	developing buds, young fruit	HQ113362

*SEP4*	2	*A. chinensis*	ripe fruit	HQ113364

**Figure 1:**
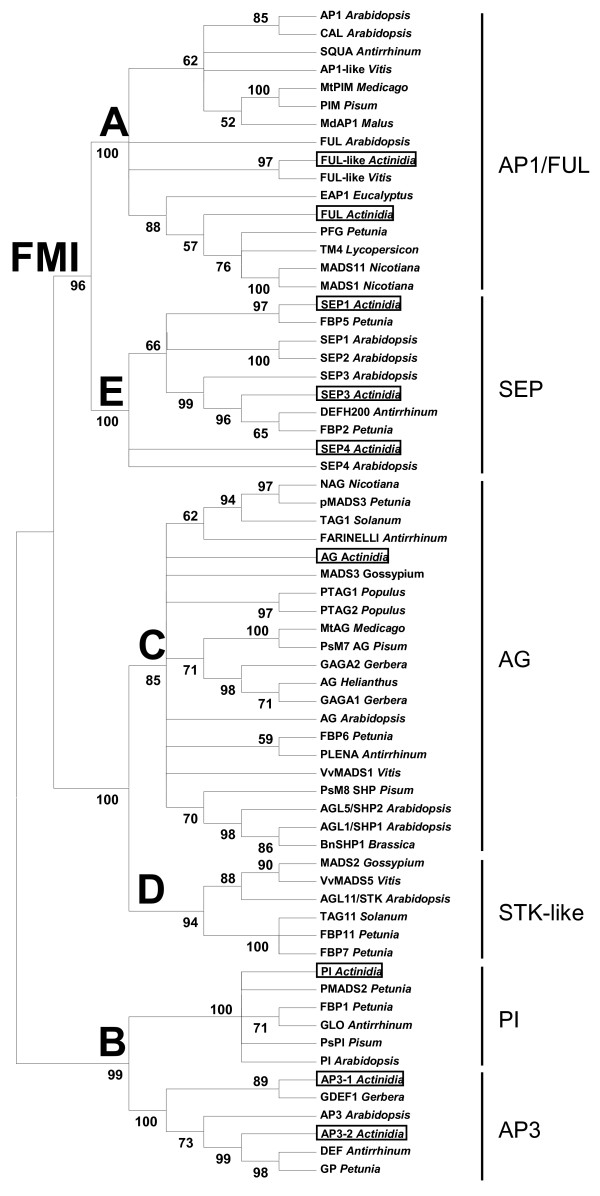
**Phylogenetic analysis of *Actinidia *flowering genes**. The MIK regions of MADS-box proteins were aligned using Clustal W (opening = 15, extension = 0.3) in Vector NTI 9.0. Phylogenetic and molecular evolutionary analyses were conducted using MEGA version 3.1 [78], using a minimum evolution phylogeny test and 1000 bootstrap replicates. Gene names shown in rectangles are *Actinidia *genes identified in this study. Class of function and floral meristem identity function (FMI) are indicated in bold.

Phylogenetic analysis further confirmed that the identified floral MADS-box genes belong to appropriate MADS-box gene families and subfamilies (Figure 1). All the predicted protein sequences of kiwifruit MADS-box genes contain the conserved MIK domains and a variable C-terminal region with conserved C-terminal motifs (Table 2). None of the identified MADS-box genes has the carboxyl-terminal CFAT/A farnesylation motif characteristic of euAP1 proteins. The predicted AG protein was clustered in the C lineage of the angiosperm AG subfamily (Figure 1), but the C and the D lineages are closely related and often difficult to distinguish. PCR amplification of the genomic DNA identified an intron located in the last codon (Additional file 1), which is characteristic of the C but not the D lineage [59].

**Table 2:** Conserved C-terminal motifs of *Actinidia *flowering genes

**Gene name**	**Conserved motif**	**Motif sequence**	**Motif consensus**	**Reference**
*FUL-like*	FUL-like	MLPWML	L/MPPWML	[66]

*FUL*	FUL-like	MPPWMF	L/MPPWML	[66]

*AP3*	paleoAP3	GCGSHDLRL	YG.HDLRLA	[85]

*AP3*	euAP3	DLTTFALLE	DLTTFALLE	[85]

*PI*	PI	FHVQPIQPNLQD	..VQP.QPNLQ.	[85]

*SEP1*	SEP	IPGWML	IPGWML	[86]

*SEP3*	SEP	MPGWLP	IPGWML	[86]

*SEP4*	SEP	IPGWML	IPGWML	[86]

### Overexrpession phenotypes of kiwifruit flowering genes

**Figure 2:**
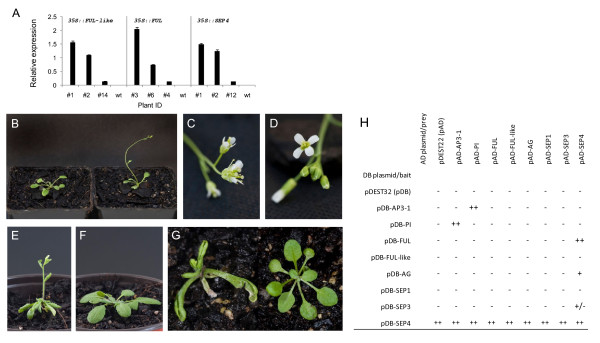
**Phenotypic analysis of transgenic *Arabidopsis *plants ectopically expressing *Actinidia *flowering genes**. A. Real-time RT-PCR analysis of transgenes in transgenic lines chosen for analysis. The expression of each gene was normalized against *ACTIN*. Error bars represent SE for three replicate reactions. B. Transgenic *Arabidopsis *plant expressing *35S::FUL-like *(right) flowered earlier than the wild type plant (left) when grown in short day conditions. C. A compound terminal flower phenotype of *35S::FUL-like *transgenic *Arabidopsis*. D. Wild type phenotype of flowers of a transgenic *Arabidopsis *plant expressing *35S::FUL*. E. Transgenic *Arabidopsis *plant expressing *35S::SEP4 *flowered early in long day conditions and produced smaller curled leaves. F. Wild type *Arabidopsis *grown as control for D. G. Transgenic *Arabidopsis *plant expressing *35S::AG *(left) and grown in short days, flowered earlier than the wild type plant (right), after producing only four curled leaves. H. Protein interactions detected by yeast-two-hybrid assay. Yeast growth, representative of protein interaction, was classified as absent (-), weak (+/-), moderate (+) or strong (++). SEP4 bait (pDB-SEP4) was excluded from analysis due to strong auto-activation.

To establish the potential role of identified genes in regulation of flowering, their cDNAs were ectopically expressed in wild type *Arabidopsis*. Among the minimum of 10 kanamycin-resistant lines per each construct, three or more were chosen for detailed analysis. In general, two of the chosen lines displayed strong phenotypes and one line was chosen that displayed a weak to moderate phenotype (Table 3; Figure 2A).

**Table 3:** Flowering time of transgenic *Arabidopsis*

***35S: :FUL-like***			
**Plant ID**	**Daylength**	**Rosette leaves**	**Days from germination**

	LD	3.8 ± 0.4	17.1 ± 0.8
#1	SD	4.0 ± 0.0	19.1 ± 0.5
	LD	3.9 ± 1.1	21.0 ± 0.7
#2	SD	4.8 ± 0.4	27.0 ± 2.9
	LD	6.8 ± 1.1	28.2 ± 1.6
#14	SD	18.6 ± 4.9	60.2 ± 4.5

***35S::FUL***			

**Plant ID**	**Daylength**	**Rosette leaves**	**Days from germination**

	LD	5.0 ± 1.4	28.7.1 ± 3.8
#3	SD	10.7 ± 4.1	47.3.1 ± 7.4
	LD	6.3 ± 0.9	30.5.1 ± 1.4
#6	SD	11.4 ± 3.9	49.2.1 ± 4.2
	LD	8.0 ± 1.3	32.6 ± 2.8
#4	SD	18.5 ± 4.5	62.0.1 ± 2.0

***35S::SEP4***			

**Plant ID**	**Daylength**	**Rosette leaves**	**Days from germination**

	LD	6.5 ± 0.8	29.8 ± 0.43
#1	SD	7.0 ± 0.8	37.7 ± 3.4
	LD	6.8 ± 0.8	27.9 ± 1.6
#2	SD	9.3 ± 1.3	37.8 ± 3.1
#12	LD	6.8 ± .7	31.0 ± 0.8
	SD	22.4 ± 2.2	64.2 ± 2.8

**Col-0**			

**Plant ID**	**Daylength**	**Rosette leaves**	**Days from germination**

	LD	8.8 ± 1.5	36.7 ± 4.2
#1	SD	29.3 ± 2.2	74.2 ± 2.8
	LD	8.2 ± 1.5	34.2 ± 2.2
#2	SD	30.2 ± 4.3	73.5 ± 5.6
#3	LD	9.2 ± 0.9	37.5 ± 5.1
	SD	30.4 ± 4.1	74.5 ± 5.8

Flowering time was recorded as number of rosette leaves and days from germination when primary inflorescence stems were 5 mm long. Three lines were chosen for detailed analysis, including two strong and a weak phenotype.

Kiwifruit *FUL-like*, when over-expressed in *Arabidopsis *Col-1 under the 35S promoter, promoted floral transition both in inductive long-day (LD) conditions and in non-inductive short-day (SD) conditions (Table 3; Figure 2B). High levels of transgene expression resulted in the terminal flower phenotype (Figure 2C). No homeotic transformation of floral whorls was detected in transgenic plants.

Kiwifruit *FUL*, when over-expressed in *Arabidopsis *Col-1 under the 35S promoter, promoted flowering but less efficiently than *FUL-like *and the flowers were indistinguishable from the wild type (Figure 2D). The ability of this construct to induce precocious flowering was dependent on day length conditions (Table 3). Constitutive over-expression of kiwifruit *SEP4 *also promoted floral transition (Table 3; Figure 2E-F). In addition, many of the plants had small and curled leaves (Figure 2F). Plants grown in short days often reverted to vegetative growth, producing aerial rosettes (data not shown). Constitutive overexpression of kiwifruit *SEP3 *had only a mild effect on the timing of floral transition in inductive LD conditions (data not shown). Ectopic expression of kiwifruit *PI *and *AP3-1 *produced plants indistinguishable from the wild type (data not shown). Constitutive over-expression of kiwifruit *AG *resulted in plants with reduced height and curled leaves, which flowered significantly earlier than the wild type in non-inductive SD conditions (Figure 2G). These plants displayed loss of inflorescence indeterminacy and homeotic modifications that resembled the phenotype of transgenic plants ectopically expressing *Arabidopsis AG *[60].

To confirm that the identified kiwifruit genes encode proteins capable of forming complexes between each other as predicted for floral MADS-box genes [28,32], a yeast-two hybrid analysis was performed. It established interactions between B class proteins AP3-1 and PI, as well as FUL and SEP4; weaker interactions were detected between AG and SEP4 and SEP3 and SEP4. No interactions were identified with FUL-like and SEP1 (Figure 2H).

### Expression patterns in vegetative and reproductive organs

**Figure 3:**
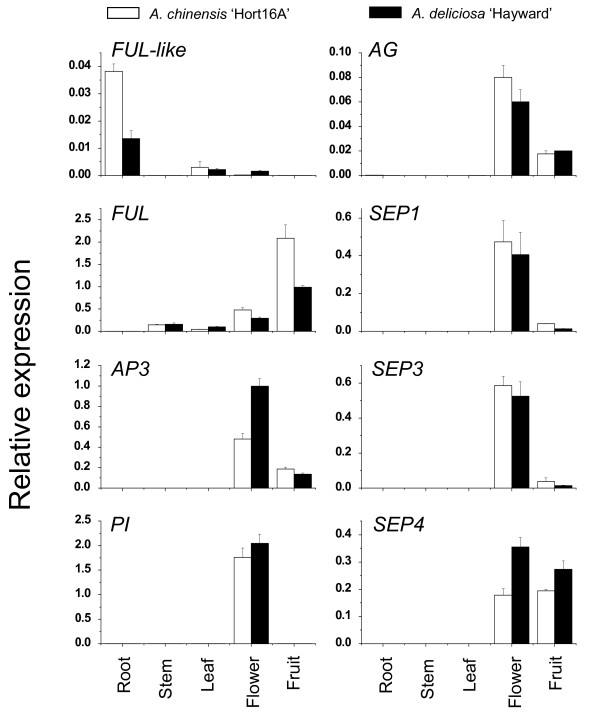
**Expression profiles of *Actinidia *flowering genes in mature plant organs**. Real-time RT-PCR analysis of the *Actinidia *flowering genes in the root, stem internode, leaf, flower and fruit of two kiwifruit cultivars, *A. chinensis *'Hort16A' (white rectangles) and *A. deliciosa *'Hayward' (black rectangles). The expression of each gene was normalized against *ACTIN*. Error bars represent SE for three replicate reactions.

MADS-box gene functions are well correlated with the expression patterns in a variety of plant species. To establish the role of identified genes in kiwifruit, their expression patterns in various vegetative and reproductive organs was interrogated by reverse transcription quantitative PCR (RT-qPCR), using two closely related kiwifruit species, a diploid *A. chinensis *and a hexaploid *A. deliciosa*, which exhibit differences in fruit characteristics, vine morphology, timing of budbreak and requirement for winter chilling. With the exception of *FUL*-like and *FUL*, expression of kiwifruit flowering genes was confined to flower and fruit tissues of both species chosen for analysis. Kiwifruit *AP3-1*, *AG*, *SEP1*, *SEP3 *and *SEP4 *were detected both in the flower and fruit tissue and *PI *was detected exclusively in flowers. Kiwifruit *FUL-like *was detected in leaf and flower tissues and was relatively highly expressed in the root. *FUL *was not detected in the root, but was detectable in vegetative shoot organs (stem and leaf) and was highly expressed in flower and particularly fruit. In general, the expression levels were relatively high compared with those of kiwifruit *ACTIN*, with the exception of *FUL-like *and *AG *(Figure 3).

### Expression domains in normal and aberrant flowers

**Figure 4:**
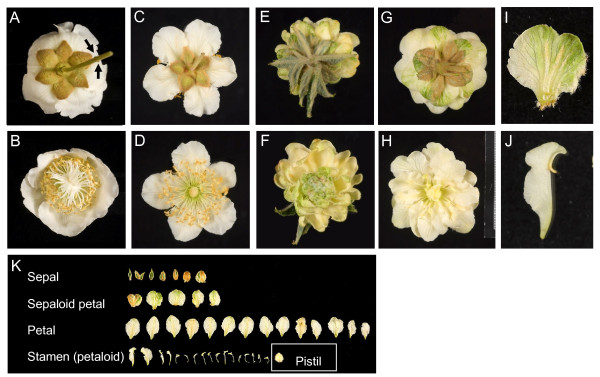
**Morphology of *Actinidia deliciosa *flowers**. A-B. *A. deliciosa *'Hayward' pistillate (female) flower with sepals, petals, stamens and ovary with a fully developed stigma. Arrows indicate small lateral bracts. C-D. *A. deliciosa *'Chieftain' staminate (male) flower with sepals, petals, stamens and a rudimentary pistil. E-F. *A. deliciosa *'Pukekohe dwarf' flower, severe phenotype, with spirally arranged large bracts in the base of the flower, multiple perianth whorls and a new flower with perianth only whorls in the centre. G-H. *A. deliciosa *'Pukekohe dwarf' flower, moderate phenotype, with small bracts, sepals, multiple whorls of petals and underdeveloped reproductive structures. I. Sepaloid petal. J. Anther-like structure fused to the petal. K. An example of sampled *A. deliciosa *'Pukekohe dwarf' floral organ tissues.

To further investigate the role of identified genes in specification of floral organ fate in kiwifruit, floral organs of normal and aberrant *A. deliciosa *flowers were analysed by RT-qPCR. *A. deliciosa *pistillate (female) flowers consist of well separated whorls, with 5-6 ovate-oblong brown sepals, 5-6 convolute white petals (Figure 4A), stamens that appear fully developed and a sub-globose, hairy ovary with numerous styles and ovules (Figure 4B). The pedicel carries two small lateral bracts (Figure 4A) that arise at very early stages of inflorescence development [61]. In some cases, lateral flowers can initiate and develop in the axils of these bracts. The staminate (male) flower is similar except for the stamens with longer filaments and larger anthers and underdeveloped ovary, which lacks styles and ovules (Figure 4C, D).

In a *A. deliciosa *mutant 'Pukekohe dwarf', which bares staminate but sterile flowers, floral organs are characterized by a transition from bracts to outer and inner perianth and underdeveloped reproductive whorls. Most severely affected flowers have multiple, spirally arranged bract and perianth whorls (Figure 4E), including intermediate floral organs (bract-like sepals, sepaloid petals). No reproductive organs are apparent and a new indeterminate flower is initiated instead (Figure 4F). Moderately affected flowers consist of better separated whorls, including bracts, sepals, petals, underdeveloped stamens and filamentous pistils (Figure 4G, H), as well as intermediate organs between each whorl, such as sepaloid outer petals (Figure 4I) and anther structures fused to the upper part of inner petals (Figure 4J). Because of the lack of sharp boundaries between 'Pukekohe dwarf' floral organs, the samples collected were bracts, sepals, sepaloid petals, petals, stamens with petaloid characteristics and the pistil-like structure (Figure 4K). Leaf tissue was also included in the analysis.

**Figure 5:**
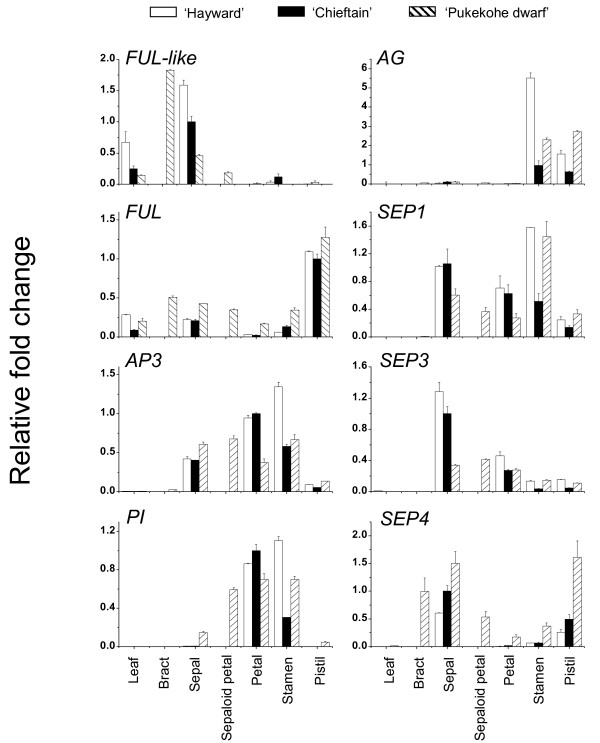
**Expression profiles of *Actinidia *flowering genes in normal and aberrant flowers**. Real-time RT-PCR analysis of the *Actinidia *flowering genes in the leaf and floral organs of *A. deliciosa *'Hayward' (female, normal), 'Chieftain' (male, normal) and 'Pukekohe dwarf' (aberrant) flowers. In addition to leaf, sepal, petal, stamen and pistil, *A. deliciosa *'Pukekohe dwarf' analysis included bracts and sepaloid petals. White rectangles, *A. deliciosa *'Hayward'; black rectangles, *A. deliciosa *'Chieftain'; grey rectangles, *A. deliciosa *'Pukekohe dwarf'. The expression of each gene was normalized against *ACTIN *and expressed as a ratio with 'Hayward' flower expression, which was set arbitrarily to 1. Error bars represent SE for three replicate reactions.

The expression patterns are presented in Figure 5. In normal flowers, *FUL-like *was expressed to high level in sepals, and moderate level in the leaf tissue. Low levels of expression were detected in other flower organs. *FUL *transcript accumulated in all tissues, but the highest accumulation was detected in the pistil tissue. *AP3-1 *was expressed in all floral organs, with higher accumulation detected in petal and stamen tissues, and *PI *was exclusively expressed in petals and stamens. *AG *accumulated in the reproductive flower organs, stamen and pistil. *SEP1 *and *SEP3 *were detected in all floral organs and *SEP4 *accumulated in sepals and pistils, with low levels of transcript detected in stamens and almost no transcript detected in petals. No major differences were apparent between male and female flowers, with the exception of female stamen tissue that accumulated higher levels of *AP3-1*, *PI*, *AG *and *SEP1 *than those detected in male stamen tissues. Similar expression domains of kiwifruit flowering genes were detected in *A. chinensis *flowers (data not shown).

In aberrant 'Pukekohe dwarf' flowers, the accumulation of kiwifruit flowering transcripts was similar to that in normal flowers, with some exceptions. *FUL-like *transcript was particularly abundant in bracts. *FUL *also accumulated in bracts to similar levels to those detected in leaves, sepals and stamens, but lower than pistil. *PI *expression domain extended across all flower organs, while being restricted to petals and stamens in normal flowers. *AG *expression was mainly confined to stamen and pistil tissue, with relative accumulation between that detected in male and female normal flowers. *SEP1 *and *SEP3 *accumulated from sepals to pistils but were absent from the leaf and bract tissue. On the other hand, *SEP4 *accumulated in the bract tissue and was also abundant in aberrant flower pistils.

### Expressions in kiwifruit emerging shoots

**Figure 6:**
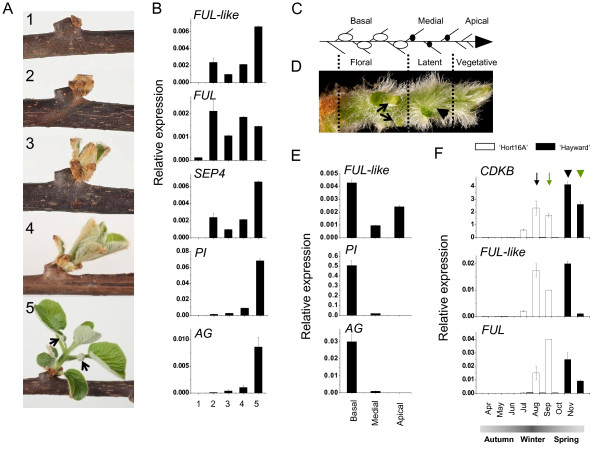
**Expression profiles of *Actinidia *flowering genes in kiwifruit emerging shoots**. A. Stages of budbreak and early shoot development in *A. chinensis *as described previously [61]. 1, dormant bud; 2, emergence of pubescent bud scales; 3, emergence of leaves; 4, unfolding of leaves and internode elongation; 5, clearly visible flower buds (arrows). Emergence of pubescent bud scales coincides with inflorescence development, when elongation of axillary meristem which gives rise to the terminal flower and initiation of bracts were detected. Leaf emergence coincides with completed sepal initiation and emergence of petal primordia. Unfolding of leaves coincides with carpel initiation [61]. B. Relative expression of kiwifruit flowering genes during shoot bud dormancy and early shoot development in *A. chinensis*. C. Schematic diagram representing the kiwifruit shoot and buds. D. Floral buds (arrows) become visible in the basal part of the stage 4 emerging shoot upon removal of leaves, but are absent from the medial part of the shoot (arrowhead). E. Relative expression of kiwifruit flowering genes in basal, medial and apical fragments of the stage 4 emerging shoot. F. Relative expression in shoot primordia during the season. Black arrow and arrowhead indicate visible emergence of pubescent bud scales in *A. chinensis *'Hort16A' and *A. deliciosa *'Hayward', respectively. Green arrow and arrowhead indicate leaf emergence in *A. chinensis *'Hort16A' and A. deliciosa 'Hayward', respectively.

Expression of kiwifruit floral genes was further analysed in emerging shoots to address their role during budbreak and early stages of inflorescence and flower development. The timing and anatomical and morphological changes during shoot development are well described [4,8,61,62] and the collected samples (Figure 6A) represented developmental stages as described using light and scanning electron microscopy by Polito and Grant [61]. Kiwifruit *FUL-like, FUL *and *SEP4 *transcripts accumulated rapidly at the time of emergence of pubescent bud scales (Figure 6B), a stage corresponding to early inflorescence development, when axillary meristem elongates and lateral bracts are initiated [61]. An increasing accumulation of *PI *and *AG *were detected from the bud scale emergence and leaf emergence stage, respectively (Figure 6B), during rapid sequential floral organ development [61]. The accumulation of *PI *and *AG *was confined to the basal part of the emerging shoot where floral differentiation takes place, and was not detected in the vegetative shoot tip (Figure 6C-E). The timing of *FUL-like *and *FUL *accumulation in the field-grown plants corresponded with initial stages of bud outgrowth in *A. chinensis *and *A. deliciosa *(Figure 6F) and was similar to the accumulation pattern of a cell cycle gene *CDKB1*, used as a marker of cell divisions [63].

## Discussion

### Kiwifruit flowering genes specify floral meristem and floral organ fates

A kiwifruit flower belongs to the regular eudicot flower type in which the floral organ identity is determined by expression and interaction of floral organ identity genes. Thus, a candidate gene approach was chosen for molecular analysis of kiwifruit flowering. Putative orthologs of genes controlling flower development were isolated and characterized from the EST collection comprising transcripts from a variety of tissues of several *Actinidia *species, including flower, developing buds and fruit [12]. The EST collection is biased towards fruit transcripts and many of the ESTs for floral organ identity were identified in fruit libraries: kiwifruit *FUL*, *AP3*, *AG*, *SEP1*, *SEP3 *and *SEP4 *were all represented with at least one sequence in a library derived from fruit transcripts (Table 1). All these genes have been confirmed as expressed in the fruit, in addition to the flower. *FUL-like *was identified from the leaf library and the presence of only one sequence correlated with its low expression levels as compared to *ACTIN*. Phylogenetic analysis and phenotypes obtained upon ectopic expression in *Arabidopsis *suggested evolutionary and functional conservation of kiwifruit flowering genes.

These data taken together with expression patterns in normal and aberrant kiwifruit flowers confirmed that the identified B-, C- and E-class genes have a role in specification of kiwifruit floral organs. The floral promotion obtained upon overexpression in *Arabidopsis*, elevated expression in 'Pukekohe dwarf' bracts and accumulation at the earliest stages of bud development strongly suggested a role for kiwifruit *FUL-like*, *FUL *and *SEP4 *in floral meristem specification. The mechanism of kiwifruit FUL-like and FUL action is unknown, but might be related to promotion or maintenance of cellular expansion and differentiation, as reported for FUL in *Arabidopsis *[64]. Expression in vegetative tissues would support the role for *FUL-like *and *FUL *genes in general cellular function. While kiwifruit *SEP4 *might perform a similar general function, it marks the inflorescence, flower and fruit development, based on the transcript absence from vegetative tissues. The increasing accumulation during shoot emergence and expression confined to reproductive organs suggested *PI *and *AG *as markers of flower differentiation.

### Is there an AP1-like gene in kiwifruit?

It is unclear if an *AP1 *orthologous gene exists in the kiwifruit genome. None of the candidate genes mined from the EST database or described previously [5] contained the carboxyl-terminal CFAT/A farnesylation motif characteristic of euAP1 proteins [65]. It is therefore possible that an unidentified kiwifruit euAP1 protein is required for sepal and petal identity. On the other hand, the role of eu*AP1 *genes in specification of sepal and petal identity in plants other than *Arabidopsis *is unclear and the concept of the A function in flower organ identity may not be universal [52,53]. In general, the role of A function genes in organ identity might simply be the result of their meristem identity function and the mutant floral organ phenotypes could be explained by incomplete transition to a floral meristem [54]. In addition, the numerous duplication events that gave rise to the angiosperm *AP1/FUL*-like gene family [44,66-68] make it difficult to identify the true orthologs across plant taxa. *FUL *genes also perform the floral meristem identity function [36,69-71] and possibly the A function [72-74], and the AP1 conserved motif and protein modification may not be necessary [75]. A possibility exists that kiwifruit *FUL-like *may have a role in sepal specification, based on its expression pattern in flowers. However, expression of *FUL-like *failed to rescue the *ap1-1 *organ identity phenotype (data not shown) and a yeast-two-hybrid assay failed to identify an interaction between FUL-like and any of the kiwifruit SEP proteins, that would have been predicted from the quartet model [28,32]. Taken together with expression in vegetative kiwifruit organs, these data suggest against the A function of kiwifruit *FUL-like*.

### *A. deliciosa *'Pukekohe dwarf' mutant - a tool to study kiwifruit flower development

Functional characterization of a gene typically includes analysis of phenotypes resulting from a mutation or ectopic expression of the gene. In kiwifruit, artificially generated mutations are difficult to generate and screen and genetic transformation is a difficult and lengthy process. However, natural genetic variants exist with altered floral development and morphology that can be utilized as a genetic tool to identify and characterize genes involved in flowering. *A. deliciosa *'Pukekohe dwarf' flower is characterized by numerous bracts preceding sepals. Given the position on the pedicel, spiral phyllotaxis and morphology significantly different from that of both leaves and sepals, bracts can be seen as modified leaves produced during the early stages of the floral transition, that arise as a result of increasing activity of floral-promoting genes. Thus, the significant up-regulation of kiwifruit *FUL-like*, *FUL *and *SEP4 *in bracts further suggests their role in establishment of floral fate, consistent with floral promotion seen upon expression of these genes in *Arabidopsis*. The other unusual feature of 'Pukekohe dwarf' flowers is the presence of multiple perianth whorls with gradual transition between floral organs and the presence of intermediate organs with combined sepal and petal or petal and anther identity. Accordingly, kiwifruit *PI *expression domain in 'Pukekohe dwarf' is extended and resembles the 'fading border' model of floral gene expression [57].

The molecular mechanisms involved in the generation of the mutation and the target genes affected in 'Pukekohe dwarf' are unknown. 'Pukekohe dwarf' is impaired in the specification of stamen/anther and pistil/carpel identity, but not in development of perianth organs, resembling the loss-of-function mutation in class C gene *AG *[13,22]. However, no major differences were detected in the expression pattern or sequence of kiwifruit *AG *between normal and aberrant flowers (Additional file 2), suggesting that a different mechanism might underlie the 'Pukekohe dwarf' phenotype. Given that the mutation likely occurs in one copy of the target gene, it is highly probable that the phenotype is the result of a gain-of-function rather than loss-of-function mutation.

### A model for kiwifruit floral organ identity

**Figure 7:**
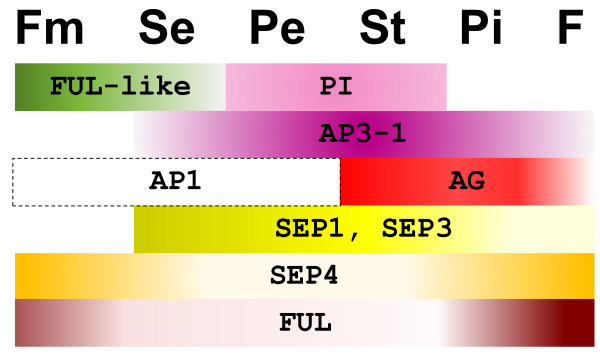
**A model for floral meristem and floral organ specification in *Actinidia***. Fm, floral meristem; Se, sepal; Pe, petal; St, stamen; Pi, pistil; F, fruit.

Based on the relative accumulation of kiwifruit MADS-box gene transcripts in the wild-type male and female flowers and 'Pukekohe dwarf' mutant flowers, a model of kiwifruit floral organ specification was proposed (Figure 7). Sharp gene expression boundaries combined with gradients of gene expression within the expression domains are responsible for the (A)BCE-like floral organ identity in the wild type, regular, four-whorled kiwifruit flower. The A function may require a yet unidentified AP1-like protein, or is derived from floral meristem specifying factors, e.g. FUL-like, FUL and SEP4 proteins; their accumulation is sufficient to promote bracts as intermediates between leaves and sepals, but additional SEP proteins (SEP1 or SEP3) are required for true sepal-identity. Extended PI expression in the mutant is the likely reason for extended petaloid features and multiple petal whorls.

## Conclusions

Over the past decades, kiwifruit has developed into an important horticultural crop, firstly in New Zealand and subsequently in other countries. Current research on kiwifruit is mainly focused around consumer-driven traits such as fruit flavour and fragrance, appearance, healthful components and convenience, but the knowledge of the genetic regulation of flowering is very scarce. This report provides a description of flower development in kiwifruit at the molecular level. It has identified genes that will be utilized as genetic markers for inflorescence and flower development and candidates that are expected to have an impact on kiwifruit growth, phase change and time of flowering.

## Methods

### Plant materials

The majority of the plant material used in this study was collected from two female cultivars that are grown commercially in New Zealand: 'Hort16A' (*A. chinensis *Planch.) and'Hayward' (*A. deliciosa *(A. Chev.) C.F. Liang et A.R. Ferguson). For expression studies in mature flowers, in addition to female *A. deliciosa *'Hayward' normal flowers, male *A. deliciosa *'Chieftain' normal flowers and *A. deliciosa *'Pukekohe dwarf' aberrant flowers were used. All kiwifruit plants were grown in the orchard under natural climatic conditions. The vines were trained on a T-bar training system and underwent pruning according to standard orchard management practice. All studies were carried out on tissue collected from at least six individual plants, with the exception of 'Pukekohe dwarf', where only one plant was available.

Root, stem, leaf, flower, and fruit tissue collection was carried out on *A. chinensis *'Hort16A' and *A. deliciosa *'Hayward' vines growing at the Plant & Food Research orchard near Kerikeri, New Zealand, during the spring and summer season of 2005-06. Mature flower organ tissues were collected from *A. deliciosa *'Hayward', 'Chieftain' and 'Pukekohe dwarf' growing in the research orchard near Te Puke, New Zealand, during the season of 2006-2007.

For expression analysis during early shoot development, kiwifruit vines (canes) were collected from *A. chinensis *'Hort16A' grown in the research orchard near Te Puke, New Zealand in autumn 2010. The canes were stored at 4°C for three weeks. Excised canes are frequently used in budbreak and flowering studies and cold storage is a standard practice that maintains buds at dormant state [76]. Bud development was initiated upon immersion of lower side of the cane in water and maintenance in 24°C and natural light. Whole dormant buds and emerging shoots were collected. For analysis in the fragments of the emerging shoot, unfolding leaves were removed prior to collection of the basal (flower-bearing), medial and apical fragments.

For seasonal expression analysis, samples were collected from autumn to late spring at monthly intervals from A*. chinensis *'Hort16A' and *A. deliciosa *'Hayward' vines growing in the research orchard near Te Puke, New Zealand, during the season of 2006-2007. Shoot primordia were dissected from axillary buds from the second, third and fourth node distal to the most distal fruit and represented the same developmental unit, starting as latent buds established during the first season that were dormant over the winter months and resumed development and outgrowth in the spring. The last two samples collected corresponded to emergence of pubescent bud scales and leaf emergence, respectively.

*Arabidopsis thaliana *'Columbia' (Col-0) ecotype plants were grown in the greenhouse under a long day (21°C, 14/10 h light/dark) or short day regime (21°C, 8/16 h light/dark).

### Phylogeny

The *Actinidia *EST database [12] was interrogated using BLAST analysis [77]. Kiwifruit EST sequences were uploaded to Vector NTI version 9.0.0 http://www.invitrogen.com. Sequence alignment was performed using Vector NTI Clustal W (opening 15, extension penalty 0.3). Phylogenetic analyses were conducted using MEGA version 3.1 [78] using a minimum evolution phylogeny test and 1000 bootstrap replicates.

### Gene isolation and vector construction

Clones containing full-length cDNA of *FUL-like*, *FUL*, *AP3*, *PI*, *SEP3 *and *SEP4 *were obtained from the Plant & Food Research *Actinidia *EST library [12]. cDNA of *FUL-like*, *FUL *and *SEP3 *were cloned as *Spe*I-*Xho*I fragments into the corresponding sites of pSAK778 binary vector [79]. cDNA of *SEP4 *was cloned as an *Asp*718-*Xba*I fragment into the corresponding sites of pART277 binary vector, produced by cloning the pART7 *Not*I CaMV35S-mcs-ocs3'cassette into the *Not*I site of pART27 [80]. cDNAs of *AP3 *and *PI*, flanked by the attB1 and attB2 sequences in pCMV-SPORT6 (Invitrogen, Life Technologies Corp.), were transferred to the Gateway-adapted pHEX2 destination binary vector [81], via the pDONR221 intermediate vector, using Gateway recombination cloning, with all reactions performed as recommended by the manufacturer (Invitrogen). Full-length cDNA copy of *AG *was isolated from *A. chinensis *flower cDNA using PCR amplification (primer sequences listed in Additional file 3), cloned into pGEM-T Easy (Promega) for sequencing purposes and further cloned as *Spe*I-*Xba*I fragment into corresponding sites of pSAK778 binary vector [79]. Construction of these plant transformation vectors placed each full-length cDNA between the CaMV 35S promoter and ocs 3' transcriptional terminator. The resulting plasmids were transformed into *Agrobacterium tumefaciens *strain GV3101 by the electroporation method.

### *Arabidopsis *transformation

*Agrobacterium tumefaciens*-mediated plant transformation was performed by the floral dipping method [82]. Seeds of transgenic plants were selected on 1/2 MS medium supplemented with kanamycin and placed in a growth room under appropriate day-length conditions.

### RNA extraction and expression studies

Total RNA was isolated from kiwifruit tissue as described by Gasic et al [83]. The concentration of RNA was determined using the NanoDrop ND-1000 Spectrophotometer (NanoDrop Technologies, Wilmington, DE). Reverse transcription (RT) was performed using RNA treated with DNase I (Invitrogen), an oligo(dT) primer and the SuperScript III reverse transcriptase (Invitrogen) according to the manufacturer's instructions. Quantifications using real-time PCR were performed with the FastStart DNA Master SYBR Green I reaction mix (Roche Diagnostics, Manheim, Germany) using the Lightcycler 1.5 instrument and the LightCycler Software version 4 (Roche Diagnostics). Primers were designed to produce amplification products within the range of 160-260 nucleotides. Primer sequences are listed in Additional file 3. The specificity of primer pairs was confirmed by melting curve analysis of PCR products and agarose gel electrophoresis followed by sequencing. Reactions were performed in triplicates and a negative water control was included in each run. Products were quantified against the standard curve using dilutions of a sample with the highest expression and the expression was normalized to kiwifruit *ACTIN *(GenBank accession number FG403300). Error bars shown in the qPCR data represent the standard error (SE) of three replicate PCR reactions.

### Yeast-two-hybrid assays

Full-length open reading frames of *Actinidia *MADS-box genes were amplified using a two-step adapter PCR strategy which incorporated the complete attB sequence (Additional file 3). The PCR fragment was recombined in the Gateway™ pDONR221 vector, resulting in an entry clone. All entry clones were verified using sequence analysis before cloning in the yeast two-hybrid GAL4 binding domain vector pDEST32 (bait) and GAL4 activating domain vector pDEST22 (prey). Bait vectors were transformed into yeast strain PJ69-4α (MATα;[84]) and prey vectors into strain PJ69-4a (MATa;[84]) and selected on SC plates lacking Leu and Trp, respectively. The baits and preys were systematically mated by spotting 5 μL droplets on top of each other on SC complete plates. After overnight incubation at 30°C, the yeast was transferred to SC plates lacking Leu and Trp to select for diploid yeast containing both plasmids, and incubated at 30°C for 2 days. This transfer was performed once more, after which the yeast was transferred to separate selection plates containing SC medium lacking Trp, Leu and His and supplemented with 0, 1, 3 or 5 mM 3-amino-1,2,4-triazole. Plates were incubated for four days at 20°C and scored for growth. The screening was performed in duplicate.

## Authors' contributions

EV-G conceived the project, acquired the funding, designed the experiments, conducted analyses and prepared the manuscript. SMM participated in *Arabidopsis *transformation and RNA extraction and expression studies. CV carried out the yeast-two-hybrid assays and contributed to the manuscript. RW participated in RNA extraction and expression studies. RHL and Y-YW participated in gene isolation and vector construction. RPH participated in the sequence alignment and has given final approval of the version to be published. All authors read and approved the final manuscript.

## Supplementary Material

Additional file 1 **Intron characteristic of *AG *C lineage, An intron located in the last codon of predicted *Actinidia AG *gene was amplified from *A. deliciosa *'Hayward' and *A. chinensis *"Hort16A'**. The presence of this intron is characteristic for the C but not the D lineage of the angiosperm AG subfamily. Three types of intron sequences were obtained from a hexaploid 'Hayward' (one 95 bp and two 188 bp in length) and one type was amplified from a diploid 'Hort16A' (193 bp). Click here for file

Additional file 2 **Nucleotide sequence alignment of *Actinidia AG *cDNA**. cDNA was amplified from *A. chinensis *'Hort16A', *A. deliciosa *'Hayward' and *A. deliciosa *'Pukekohe dwarf' flower cDNA. The *A. arguta AG *sequence was obtained from the *Actinidia *EST database. Click here for file

Additional file 3 **Nucleotide sequence of oligonucleotides used in this study**. Click here for file
