# Supplementary material for: Identification and characterization of flowering genes in kiwifruit: sequence conservation and role in kiwifruit flower development
Source: BMC Plant Biol. 2011 Apr 27;11:72. doi: 10.1186/1471-2229-11-72 (PMC3103426; doi:10.1186/1471-2229-11-72)
Supplement: Additional file 1 — Intron characteristic of AG C lineage, An intron located in the last codon of predicted Actinidia AG gene was amplified from A. deliciosa 'Hayward' and A. chinensis "Hort16A'. The presence of this intron is characteristic for the C but not the D lineage of the angiosperm AG subfamily. Three types of intron sequences were obtained from a hexaploid 'Hayward' (one 95 bp and two 188 bp in length) and one type was amplified from a diploid 'Hort16A' (193 bp). [file 1471-2229-11-72-S1.PDF]

Additional file 1

A

```
… TAT TCT CGC CAA GAC CAA ACA GTC CTT CAG CTA GTA TGA AGGTCCAGCTATTTATGGAAAGTGCTAGCTGAAGCTTT

                                          Intron
```

B

```
Hayward1    (1) GTA--------------CGTC-------------------------------------------------------------------------------
Hayward2    (1) GTACGTATTTTA-----CGTCGCTTTAATTTATGCTTTTCTTTTTCGCATGGCACATATTAACGTCGAAAATATTGAAATGAAATAAATTAAGTGCAGCC
Hayward3    (1) GTACGTATTTTA-----CGTCGCTTTAATTTATGCTTTTCTTTTTCGCATGGCACATATTAACGTCGAAAATATTGAAATGAAATAAATTAAGTGCAGCC
Hort16A     (1) GTACGTATTTTATTTTACGTCGCTTTAATTTATGCTTTTCTTTTTCGCATGGCACATATTAACGTCGAAAAAATTGAAATGAAATAAATTAAGTGCAGCC

Hayward1    (8) ---TTTCATATGCATATCTGATATAAAAGAGATGGAAGAAAAAA--TAAAGCACAAACAAACGAATCTGAAATAAGTTTCCGTATTTTAGCAG
Hayward2   (96) TAATTTCATATGCATATTTGATAGAAAAGAGATGGAAGAAAAAAAATAAAGCACAAACAAACGAATCTGAAGTAAGTTTTCGTATTTTAGCAG
Hayward3   (96) TAATTTCATATGCATATTTGATAGAAAAGAGATGGAAGAAAAAAAATAAAGCACAAACAAACGATTCTGAAATAAGTTTTCGTATTTTAGCAG
Hort16A   (101) TAATTTCATATGCATATTTGATAGAAAAGAGATGGAAGAAAAAAAATAAAGCACAAACAAACGAATCTGAAATAAGTTTTCGTATTTTAGCAG
```
