# Supplementary material for: Identification and characterization of flowering genes in kiwifruit: sequence conservation and role in kiwifruit flower development
Source: BMC Plant Biol. 2011 Apr 27;11:72. doi: 10.1186/1471-2229-11-72 (PMC3103426; doi:10.1186/1471-2229-11-72)
Supplement: Additional file 2 — Nucleotide sequence alignment of Actinidia AG cDNA. cDNA was amplified from A. chinensis 'Hort16A', A. deliciosa 'Hayward' and A. deliciosa 'Pukekohe dwarf' flower cDNA. The A. arguta AG sequence was obtained from the Actinidia EST database. [file 1471-2229-11-72-S2.PDF]

Additional file 2

```
                                51                                               100
      A.arguta    (1)  ATGGAGGGCTCTCTCTCTCCTCAGAAGAAAATGGGAGGCAGGGGGAAGAT
   A.chinensis    (1)  ATGGAGGGCTCTCTCTCTCCTCAGAAGAAAATGGGAGGAAGGGGGAAGAT
   A.deliciosa    (1)  ATGGAGGGCTCTCTCTCTCCTCAGAAGAAAATGGGAGGAAGGGGGAAGAT
Pukekohe dwarf    (1)  ATGGAGGGCTCTCTCTCTCCTCAGAAGAAAATGGGAGGAAGGGGGAAGAT
                       51                                               100
      A.arguta   (51)  CGAGATCAAGCGGATCGAAAACACGACGAATCGCCAGGTCACCTTTTGTA
   A.chinensis   (51)  CGAGATCAAGCGGATCGAAAGCACGACGAACCGCCAGGTCACCTTTTGTA
   A.deliciosa   (51)  CGAGATCAAGCGGATCGAAAACACGACGAATCGCCAGGTCACCTTTTGTA
Pukekohe dwarf   (51)  CGAGATCAAGCGGATCGAAAACACGACGAATCGCCAGGTCACCTTTTGTA
                       101                                              150
      A.arguta  (101)  AGCGCCGTAATGGTTTGCTTAAGAAAGCCTATGAGCTCTCTGTCCTCTGT
   A.chinensis  (101)  AGCGCCGTAATGGTTTGCTTAAGAAAGCCTATGAGCTCTCTGTCCTCTGT
   A.deliciosa  (101)  AGCGCCGTAATGGTTTGCTTAAGAAAGCCTATGAGCTCTCTGTCCTCTGT
Pukekohe dwarf  (101)  AGCGCCGTAATGGTTTGCTTAAGAAAGCCTATGAGCTCTCTGTCCTCTGT
                       151                                              200
      A.arguta  (151)  GATGCTGAAGTTGCTCTCATTGTCTTCTCCACTCGCGGCCGCCTTTACGA
   A.chinensis  (151)  GATGCTGAAGTTGCTCTCATTGTCTTCTCCACTCGCGGCCGCCTTTACGA
   A.deliciosa  (151)  GATGCTGAAGTTGCTCTCATTGTCTTCTCCACTCGCGGCCGCCTTTACGA
Pukekohe dwarf  (151)  GATGCTGAAGTTGCTCTCATTGTCTTCTCCACTCGCGGCCGCCTTTACGA
                       201                                              250
      A.arguta  (201)  GTACGCCAACAACAGTGTGAAAGGAACCATTGAAAGGTACAAGAAGGCAA
   A.chinensis  (201)  GTACGCCAACAACAGTGTGAAAGGAACCATTGAAAGGTACAAGAAGGCAA
   A.deliciosa  (201)  GTACGCCAACAACAGTGTGAAAGGAACCATTGAAAGGTACAAGAAGGCAA
Pukekohe dwarf  (201)  GTACGCCAACAACAGTGTGAAAGGAACCATTGAAAGGTACAAGAAGGCAA
                       251                                              300
      A.arguta  (251)  GCTCAGGCTCCTCTAATACTGGATCGGTTTCTGAACTAAATGCTCAGTTT
   A.chinensis  (251)  GCTCAGGCTCCTCTAATACTGGGTCGGTTTCTGAACTAAATGCTCAGTTT
   A.deliciosa  (251)  GCTCAGGCTCCTCTAATACTGGATCGGTTTCTGAACTAAATGCTCAGTTT
Pukekohe dwarf  (251)  GCTCAGGCTCCTCTAATACTGGATCGGTTTCTGAACTAAATGCTCAGTTT
                       301                                              350
      A.arguta  (301)  TATCAGCAAGAAGCCGCTAAATTGCGCGTGCAAATTAATAATTTGCAGAG
   A.chinensis  (301)  TATCAGCAAGAAGCCGCCAAATTGCGCGTGCAAATTAATAATTTGCAGAG
   A.deliciosa  (301)  TATCAGCAAGAAGCCGCCAAATTGCGCGTGCAAATTAATAATTTGCAGAG
Pukekohe dwarf  (301)  TATCAGCAAGAAGCCGCCAAATTGCGCGTGCAAATTAATAATTTGCAGAG
                       351                                              400
      A.arguta  (351)  CTCAAACAGCCACATGCACGGCGATTCTCTGAGCTCTATGTCTCTCAGAG
   A.chinensis  (351)  CTCAAACAGCCACATGCGCGGCGAGTCTCTGAGCTCTTTGTCTCTCAGGG
   A.deliciosa  (351)  CTCAAACAGCCACATGCGCGGGGAGTCTCTGAGCTCTTTGTCTCTCAGGG
Pukekohe dwarf  (351)  CTCAAACAGCCACATGCGCGGGGAGTCTCTGAGCTCTTTGTCTCTCAGGG
                       401                                              450
      A.arguta  (401)  AACTCAAGAACCTCGAGGGTCGTTTAGAGAGAGGCATTAGCAGGATCCGC
   A.chinensis  (401)  AGCTCAAGAACCTCGAGGGTCGTTTAGAGAGAGGCATTAGCAGGATCCGC
   A.deliciosa  (401)  AACTCAAGAACCTCGAGGGTCGTTTAGAGAGAGGCATTAGCAGGATCCGC
Pukekohe dwarf  (401)  AACTCAAGAACCTCGAGGGTCGTTTAGAGAGAGGCATTAGCAGGATCCGC
                       451                                              500
      A.arguta  (451)  TCCAAAAAGAATGAGCTTTTGTTTGCTGAAATCGAGCTTATGCAAAAGAG
   A.chinensis  (451)  TCCAAAAAGAATGAGCTTTTGTTTGCTGAAATCGAGCTTATGCAAAAGAG
   A.deliciosa  (451)  TCCAAAAAGAATGAGCTGTTGTTTGCTGAAATCGAGCTTATGCAAAAGCG
Pukekohe dwarf  (451)  TCCAAAAAGAATGAGCTTTTGTTTGCTGAAATCGAGCTTATGCAAAAGAG
                       501                                              550
      A.arguta  (501)  GGAAGCGTATTTGCCTCAGAATACCCAATGCCTCAGAGCAAAGATAGCTG
   A.chinensis  (501)  GGAAGTCGACTTGCATCAGGATAACCAGTACCTGACAGCAAAGATAGCTG
   A.deliciosa  (501)  GGAAGTCGACTTGCATCGGGATAACCAGTACCTGAGAGCAAAGATAGCTG
Pukekohe dwarf  (501)  GGAAGTCGACTTGCATCAGGATAACCAGTATCTGAGAGCAAAGATAGCTG
                       551                                              600
      A.arguta  (551)  AGGGTGAGAGAGTGCAGCAGATGAATTTGATGCCCGCAGGGTCCGACTAT
   A.chinensis  (551)  AGGGTGAGAGAGTGCAGCAGATGAATTTGATGCCCGGAGGGTCTGAGTAT
   A.deliciosa  (551)  AGGGTGAGAGAGTGCAGCAGATGAATTTGATACCCGGAGGGTCTGACTAT
Pukekohe dwarf  (551)  AGGGTGAGAGAGTGCAGCAGATGAATTTGATGCCCGGAGGGTCTGAGTAT
                       601                                              650
      A.arguta  (601)  GAGTTGATGCAGCAGCCTCCGTCATTAGACACTCGCAATTATCTTCAAGT
   A.chinensis  (601)  GAGTTGATGCAGC---CTCCGTCATTCGACACTCGCAATTATCTTCAAGT
   A.deliciosa  (601)  GAGTTGATGCAGC---CTCCGTCATTCGACACTCGCAATTATCTTCAAGT
Pukekohe dwarf  (601)  GAGTTGATGCAGC---CTCCGTCATTCGACACTCGCAATTATCTTCAAGT
                       651                                              700
      A.arguta  (651)  GGATGGCCGGCTGCAATCCGATCATAATTCGTATTCTCGCCAAGACCAAA
   A.chinensis  (648)  GGATGGCCGGCTGCAATCCGATCATAATTCGTATTCTCGCCAAGACCAAA
   A.deliciosa  (648)  GGATGGCCGGCTGCAATCCGATCATAATTCGTATTCTCGCCAAGACCAAA
Pukekohe dwarf  (648)  GGATGGCCGGCTGCAATCCGATCATAATTCGTATTCTCGCCAAGACCAAA
                       701                720
      A.arguta  (701)  CAGTCCTTCAGCTAGTATGA
   A.chinensis  (698)  CAGTCCTTCAGCTAGTATGA
   A.deliciosa  (698)  CAGTCCTTCAGCTAGTATGA
Pukekohe dwarf  (698)  CAGTCCTTCAGCTAGTATGA
```
