# Supplementary material for: Identification and characterization of flowering genes in kiwifruit: sequence conservation and role in kiwifruit flower development
Source: BMC Plant Biol. 2011 Apr 27;11:72. doi: 10.1186/1471-2229-11-72 (PMC3103426; doi:10.1186/1471-2229-11-72)
Supplement: Additional file 3 — Nucleotide sequence of oligonucleotides used in this study. [file 1471-2229-11-72-S3.PDF]

Additional file 3

RT-qPCR primers

Gene	Primer	Oligonucleotide sequence
FUL-like	Forward	5’-GCTTCAACATGTGGAGCAACAGA-3’
	Reverse	5’-GAGGCCTTGGTGGTTGAGGTAAC-3’
FUL	Forward	5’-CACAGCCATTGCAGTCCTTCAACA-3’
	Reverse	5’-CATTTGAGCCTGCCCTTAACAACG-3’
AP3	Forward	5’-AACTTGGAGGAAAGGCATGGAAA-3’
	Reverse	5’-ACTTAAGCAAGGCGAAGATCGTG-3’
PI	Forward	5’-AGAGCTCATGGCCTTGGAAGAAG-3’
	Reverse	5’-GTCCTGCAAATTAGGCTGGATCG-3’
AG	Forward	5’-AGGATCCGCTCCAAAAAGAATGA-3’
	Reverse	5’-TGTTTGGTCTTGGCGAGAATACG-3’
SEP1	Forward	5’-ACAAAGACCCAATCTATGCTG-3’
	Reverse	5’-TGCTGGGCATAGGAAGTACAT-3’
SEP3	Forward	5’-ACACGGACCCAGTACATGCTT-3’
	Reverse	5’-CCTGAGGTTGAGCTGGTTGTC-3’
SEP4	Forward	5’-ATGCTAGCTGAGTCCAACAAA-3’
	Reverse	5’-TCGGACTGGGCAGGAACGTGAT-3’
ACTIN	Forward	5’-CCAAGGCCAACAGAGAGAAG-3’
	Reverse	5’-GACGGAGGATAGCATGAGGA-3’

Oligonucleotides used to amplify kiwifruit AG full-length cDNA and intron sequence

Gene	Primer	Oligonucleotide sequence
AG	Forward-SpeI	5’-GGACTAGTATGGAGGGCTCTCTCTCT-3’
	Reverse-XbaI	5’-CGTCTAGATCATACTAGCTGAAGGACT-3’
AG	Intron-F	5’-CCGTCATTAGACACTCGCAATTA-3’
	Intron-R	5’-AAAGCTTCAGCTAGCACTTTCCA-3’

Oligonucleotides used for yeast-two-hybrid assay

attB1-AP3-1:	5’-AAAAAGCAGGCTTCATGGGGCGTGGAAAGATC-3’
attB2-AP3-1:	5’-AGAAAGCTGGGTCTTAAGCAAGGCGAAGATCG-3’
attB1-PI:	5’-AAAAAGCAGGCTTCATGGGGAGAGGGAAGATA-3’
attB2-PI:	5’-AGAAAGCTGGGTCTCAAATCCTGTCCTGCAAATTAG-3’
attB1-FUL:	5’-AAAAAGCAGGCTTCATGGGGAGAGGGAGAGTG-3’
attB2-FUL:	5’-AGAAAGCTGGGTCCTATCCATTGATGTGCCG-3’
attB1-FUL-like:	5’-AAAAAGCAGGCTTCATGGGGCGGGGTCGGGTT-3’
attB2-FUL-like:	5’-AGAAAGCTGGGTCTTATTGATTCACCTGGTGGAG-3’
attB1-AG:	5’-AAAAAGCAGGCTTCATGGAGGGCTCTCTCTCT-3’
attB2-AG:	5’-AGAAAGCTGGGTCTCATACTAGCTGAAGGACTG-3’
attB1-SEP1:	5’-AAAAAGCAGGCTTCATGGGGAGGGGGAGAGTG-3’
attB2-SEP1:	5’-AGAAAGCTGGGTCTCAAAGCATCCACCCAGG-3’
attB1-SEP3:	5’-AAAAAGCAGGCTTCATGGGGAGAGGTCGGGTT-3’
attB2-SEP3:	5’-AGAAAGCTGGGTCTCATGGCAACCACCCTGG-3’
attB1-SEP4:	5’-AAAAAGCAGGCTTCATGGGGAGAGGGAGAGTG-3’
attB2-SEP4:	5’-AGAAAGCTGGGTCTTAGAGCATCCACCCTGG-3’
attB1 adapter:	5’-GGGGACAAGTTTGTACAAAAAAGCAGGCT-3’
attB2-adapter:	5’-GGGGACCACTTTGTACAAGAAAGCTGGGT-3’
